# Over-Detection of Melanoma-Suspect Lesions by a CE-Certified Smartphone App: Performance in Comparison to Dermatologists, 2D and 3D Convolutional Neural Networks in a Prospective Data Set of 1204 Pigmented Skin Lesions Involving Patients’ Perception

**DOI:** 10.3390/cancers14153829

**Published:** 2022-08-07

**Authors:** Anna Sophie Jahn, Alexander Andreas Navarini, Sara Elisa Cerminara, Lisa Kostner, Stephanie Marie Huber, Michael Kunz, Julia-Tatjana Maul, Reinhard Dummer, Seraina Sommer, Anja Dominique Neuner, Mitchell Paul Levesque, Phil Fang Cheng, Lara Valeska Maul

**Affiliations:** 1 Department of Dermatology, University Hospital of Basel, 4031 Basel, Switzerland; 2 Department of Dermatology, University Hospital of Zurich, 8091 Zurich, Switzerland; 3 Faculty of Medicine, University of Zurich, 8006 Zurich, Switzerland

**Keywords:** smartphone, mobile health application, melanoma, early detection, over-detection, diagnostic accuracy

## Abstract

**Simple Summary:**

Early detection and resection of cutaneous melanoma are crucial for a good prognosis. However, visual distinction of early melanomas from benign nevi remains challenging. New artificial intelligence-based approaches for risk stratification of pigmented skin lesions provide screening methods for laypersons with increasing use of smartphone applications (apps). Our study aims to prospectively investigate the diagnostic accuracy of a CE-certified smartphone app, SkinVision^®^, in melanoma recognition. Based on classification into three different risk scores, the app provides a recommendation to consult a dermatologist. In addition, both patients’ and dermatologists’ perspectives towards AI-based mobile health apps were evaluated. We observed that the app classified a significantly higher number of lesions as high-risk than dermatologists, which would have led to a clinically harmful number of unnecessary excisions. The diagnostic performance of the app in dichotomous classification of 1204 pigmented skin lesions (risk classification for nevus vs. melanoma) remained below advertised rates with low sensitivity (41.3–83.3%) and specificity (60.0–82.9%). The confidence in the app was low among both patients and dermatologists, and no patient favored an assessment by the app alone. Although smartphone apps are a potential medium for increasing awareness of melanoma screening in the lay population, they should be evaluated for certification with prospective real-world evidence.

**Abstract:**

The exponential increase in algorithm-based mobile health (mHealth) applications (apps) for melanoma screening is a reaction to a growing market. However, the performance of available apps remains to be investigated. In this prospective study, we investigated the diagnostic accuracy of a class 1 CE-certified smartphone app in melanoma risk stratification and its patient and dermatologist satisfaction. Pigmented skin lesions ≥ 3 mm and any suspicious smaller lesions were assessed by the smartphone app SkinVision^®^ (SkinVision^®^ B.V., Amsterdam, the Netherlands, App-Version 6.8.1), 2D FotoFinder ATBM^®^ master (FotoFinder ATBM^®^ Systems GmbH, Bad Birnbach, Germany, Version 3.3.1.0), 3D Vectra^®^ WB360 (Canfield Scientific, Parsippany, NJ, USA, Version 4.7.1) total body photography (TBP) devices, and dermatologists. The high-risk score of the smartphone app was compared with the two gold standards: histological diagnosis, or if not available, the combination of dermatologists’, 2D and 3D risk assessments. A total of 1204 lesions among 114 patients (mean age 59 years; 51% females (55 patients at high-risk for developing a melanoma, 59 melanoma patients)) were included. The smartphone app’s sensitivity, specificity, and area under the receiver operating characteristics (AUROC) varied between 41.3–83.3%, 60.0–82.9%, and 0.62–0.72% according to two study-defined reference standards. Additionally, all patients and dermatologists completed a newly created questionnaire for preference and trust of screening type. The smartphone app was rated as trustworthy by 36% (20/55) of patients at high-risk for melanoma, 49% (29/59) of melanoma patients, and 8.8% (10/114) of dermatologists. Most of the patients rated the 2D TBP imaging (93% (51/55) resp. 88% (52/59)) and the 3D TBP imaging (91% (50/55) resp. 90% (53/59)) as trustworthy. A skin cancer screening by combination of dermatologist and smartphone app was favored by only 1.8% (1/55) resp. 3.4% (2/59) of the patients; no patient preferred an assessment by a smartphone app alone. The diagnostic accuracy in clinical practice was not as reliable as previously advertised and the satisfaction with smartphone apps for melanoma risk stratification was scarce. MHealth apps might be a potential medium to increase awareness for melanoma screening in the lay population, but healthcare professionals and users should be alerted to the potential harm of over-detection and poor performance. In conclusion, we suggest further robust evidence-based evaluation before including market-approved apps in self-examination for public health benefits.

## 1. Introduction

Cutaneous melanoma is one of the most aggressive cancers in humans and thus remains a major clinical challenge [1]. The incidence of melanoma in Western populations has increased rapidly over recent decades [2]. Despite a significant improvement in prevention, diagnosis, and treatment, cutaneous melanoma is still associated with a high mortality and morbidity rate [3,4,5]. Missing awareness about skin cancer prevention and the medical need for better techniques to distinguish nevi from early melanomas play a relevant role. Early detection and resection of melanoma are crucial for improving patient outcomes [6,7]. Hence, the rise of artificial intelligence (AI) has led to the hope of novel patient-autonomous melanoma diagnosis. Computer-aided diagnostic tools have also been developed to enable early detection of melanoma by laypersons in the public.

Through digitalization and the resulting widespread access to smartphones, most adults in the world own a smartphone and use applications (apps). While 92% of the adult population in the U.S. owned a mobile phone in 2015 [8], up to 97% had a mobile phone in 2021 [9]. Thereby, the use of mobile health (mHealth) apps has recently risen considerably to provide independent health care time and location [10]. Between 2014–2017, 235 new apps in the field of dermatology were registered representing a growth of 80.8% [11], while melanoma-related apps increased by 55.8% [12]. Aside from the main functionality as a source of information or awareness about melanoma, prevention, and skin self-surveillance strategies, the apps increasingly provide diagnostic and monitoring capabilities for pigmented skin lesions [13,14].

Apps using machine learning algorithms to provide risk stratification have opened new opportunities for the detection of melanoma by laypersons, as they offer immediate support in the risk classification of pigmented skin lesions and whether they require further medical advice [15]. Currently, only the SkinVision^®^ and TeleSkin skinScan apps constitute CE (Conformité Européenne) certified medical products in Europe [15]. Furthermore, only one smartphone app, the DermaCompare app (Emerald Medical Applications, Israel), has been approved by the United States Food and Drug Administration (FDA) so far [16]. However, this smartphone app is considered a CE class 1 medical device (self-certified) and is not approved for classifying skin lesions directly.

The smartphone app SkinVision^®^ is a CE class 1 (self-certified) certified medical product [17] and the most downloaded app of all melanoma screening apps in the Android store [12], reaching approximately 900,000 users in 2018 [18]. SkinVision^®^ [19] indicated promising results with high sensitivity and specificity by evaluating the diagnostic accuracy of this mHealth app in risk classification of skin lesions based on machine learning algorithms in a retrospective study [20]. However, some experts criticize that the reported sensitivity (95.1%) and specificity (78.3%) of the SkinVision^®^ app were probably overestimated in the released studies based on the nature of the study design and sampling errors [21].

If mHealth apps have high diagnostic accuracy in distinguishing benign nevi from malignant lesions, there is great potential in supporting individuals with suspicious pigmented skin lesions in their decision to consult a dermatologist or in reducing fear of skin cancer [22]. However, low diagnostic accuracy carries the risk of misinterpretation by AI, particularly over-detection by mistakenly diagnosing melanoma and associated user anxiety, as well as missing melanoma, which leads to fatal consequences. These concerns arise from several studies that have demonstrated poor diagnostic accuracy of smartphone apps for melanoma detection compared to dermatologists [23,24,25].

In addition to the diagnostic accuracy of smartphone apps, knowledge about laypersons’ willingness to use mHealth apps and their potential trust in these technologies is relevant. Therefore, healthcare professionals’ and laypersons’ personal experiences, expectations, and concerns regarding the use of AI in melanoma detection need to be analyzed. Certain barriers to mHealth apps may reduce widespread acceptance by laypersons including consideration about privacy, costs, and ethics, as well as fears about quality and reliability [26,27,28,29]. Besides critical patients’ perspectives regarding mHealth apps and AI in melanoma detection, beneficial attitudes are based on faster diagnosis, access to care, usability, and support for physicians [26,27,30,31].

To date, there are no prospective validation studies of this CE-certified melanoma detection app comparing the risk assessments with those of dermatologists and class 1 CE-certified 2D and 3D total body photography (TBP) devices alongside histopathology. The smartphone app SkinVision^®^ provides only macroscopic close-up images of the skin, while the deep-learning algorithm of the 2D and 3D convolutional neural networks (CNN) uses dermoscopic images. Data evaluating the smartphone app by patients and physicians are also sparse. Our study aims to assess the diagnostic accuracy of the CE-certified smartphone app SkinVision^®^ in melanoma detection in a real-world setting and to provide an insight into healthcare professionals’ and laypersons’ evaluation of mHealth apps for melanoma screening.

## 2. Materials and Methods

### 2.1. Study Design and Participants

We performed this prospective, single-center, comparative observational cohort study at the Department of Dermatology at the University Hospital Basel in Switzerland between January 2021 to June 2021. Seven dermatologists (beginners: <2 years professional experience, n = 4; intermediate: 2–5 years professional experience, n = 1; experts: >5 years professional experience, n = 2), as well as 114 patients were included in the study with an age of ≥18 years with a high risk for developing a primary cutaneous malignant melanoma defined as previous melanoma (including melanoma in situ), ≥100 melanocytic nevi, ≥5 atypical nevi, strong family history for melanoma, diagnosis of dysplastic nevus syndrome, or known CDKN2A mutation. Exclusion criteria were acute psychiatric illness or acute crisis and Fitzpatrick sign type V-VI.

### 2.2. Procedures

In the first step, the patients signed the informed consent after they were informed by a dermatologist about the study design and the following examinations. Subsequently, the dermatologist obtained a medical history, including a history of melanoma to enable classification into the different risk groups. The classification of all high-risk patients into further risk cohorts are defined by how often the patients require skin cancer screening. Based on this, we have classified our melanoma patients as high risk, as they come to dermatological control every 3–12 months according to the corresponding AJCC stage. Patients without melanoma in their own medical history are seen every 12 months.

All study participants underwent a standard skin cancer screening by a dermatologist (Supplementary Figure S1). The pigmented skin lesions were assessed with dermatoscopy and classified as either benign or malignant. The dermatologist defined pigmented skin lesions that were suspected melanoma and indicated whether lesions smaller than 3 mm should be classified with the different AI modalities due to suspicion. The regular skin cancer screening was performed before any AI-based risk assessments were achieved, so the dermatologists had no knowledge about AI classification of the melanocytic lesions up to this point. In the next step, all melanocytic nevi ≥ 3 mm or any smaller suspicious melanocytic lesions were assessed using an iOS-based iPhone SE smartphone equipped with a 12-megapixel camera and the app SkinVision^®^ (SkinVision^®^ B.V., Amsterdam, the Netherlands, App-Version 6.8.1), which is based on a machine-learning algorithm classifying pigmented lesions into low, medium, and high risk for melanoma. Only the lesions classified as high-risk—indicating a recommendation to consult a dermatologist—were recorded. Afterwards, patients received a whole-body screening using the 2D automated total body mapping (ATBM^®^) master of FotoFinder (FotoFinder ATBM^®^ Systems GmbH, Bad Birnbach, Germany, Version 3.3.1.0, risk scoring with MoleAnalyzer Pro), and the 3D TBP Vectra^®^ WB360 System (Canfield Scientific, Parsippany, NJ, USA, Version 4.7.1, risk scoring with DEXI) which was performed by medical students and a study nurse. Subsequently, all lesions over 3 mm or any smaller suspicious lesions were assessed by the 2D and 3D AI devices using their dermoscopes (dermoscope medicam 1000 (FotoFinder ATBM^®^) and VISIOMED^®^ D200evo dermoscope (Vectra^®^ WB360)). For dermoscopic imaged melanocytic nevi, we obtained AI-based risk scores between 0.0–1.0 for 2D FotoFinder ATBM^®^ and 0.0–10.0 for 3D TBP Vectra^®^ WB360. FotoFinder’s ATBM^®^ master creates a 2D-image of the entire surface of the patient using 20 photos taken from 8 different parts of the body, while Vectra^®^ WB360 generates a 3D-image using 92 individual photos that are converted into a 3D Avatar. Finally, the dermatologists assessed the skin lesions a second time with knowledge of the AI risk assessment scores of 2D FotoFinder ATBM^®^ and 3D TBP Vectra^®^ WB360. The patient was informed about the lesions with suspicion for melanoma by a dermatologist and about the 2D and 3D CNN classifications above the study-defined cut-off scores.

The indication for excision of suspicious lesions was based on the dermatologists’ suspicion for melanoma and/or malignancy risk assessment scores of the 2D imaging tool FotoFinder ATBM^®^ and/or 3D imaging tool Vectra^®^ WB360 indicating values over the study-defined cut-off score (>0.5 resp. >5.0). The patient was informed which lesions would be removed with the appropriate rationale. A maximum of two excisions were performed per visit and per patient due to ethical reasons, with an exception for further biopsies in cases of high-grade suspicion of malignancy by the dermatologist or patient request. The reference standard for a benign lesion was either the histological diagnosis (no melanoma) or the combination of the dermatologist’s evaluation (benign lesion) plus the AI scores of two independent medical devices (FotoFinder ATBM^®^ and Vectra^®^ WB360) each below the cut-off score.

After all the assessments, the participating patients completed an anonymous 9-item questionnaire that surveyed their attitudes, personal preferences, and concerns about the use of AI in melanoma detection compared with skin cancer screenings by a dermatologist in addition to sociodemographic data. The dermatologists completed a 2-item questionnaire assessing their attitudes toward the smartphone app in melanoma screening, which was answered specifically for each of the 114 included patients. Neither the patients nor dermatologists were informed about the result of the app’s risk assessment due to the extremely high number of false-positive findings to prevent response bias. Our survey was designed de novo after literature research. All answers were optional. The questionnaire was available in German, English and French and included binary questions (yes/no), multiple choice questions and visual analogue scales (VAS) with scores from 0–10.

### 2.3. Statistical Analysis

Comparison of continuous variables was tested with the Wilcoxon rank test. Comparison of categorical variables was tested with Fishers Exact test. A *p*-value of less than 0.05 was deemed as significant. Receiver Operating Characteristics (ROC) analysis was used to assess the performance of the SkinVision^®^ app against the combined evaluation of FotoFinder ATBM^®^, Vectra^®^ WB360, and the dermatologist and the SkinVision^®^ app against histology. All analyses were performed with R (version 4.1) and visualized with ggplot2.

### 2.4. Ethics

The study was approved by the local ethics committee (22020-02482), registered with ClinicalTrials.gov (NCT04605822), and was conducted in compliance with the Declaration of Helsinki and Good Clinical Practice GCP-rules.

## 3. Results

### 3.1. Study Population

Overall, data from 114 patients were analyzed, including 55 patients at high-risk for developing a melanoma (mean age 55 years (age range): 22–85), 47% females) and 59 melanoma patients (mean age 60 years (age range: 29–81), 54% females) (Table 1). The family history for melanoma was positive in 19% of the melanoma patients and in 56% of the non-melanoma patients. Most of the patients used sunscreen SPF 30–50 (51% of melanoma patients vs. 62% of patients at high-risk for melanoma) and had previous sunburns in childhood (54% resp. 69%).

**Figure 1:**
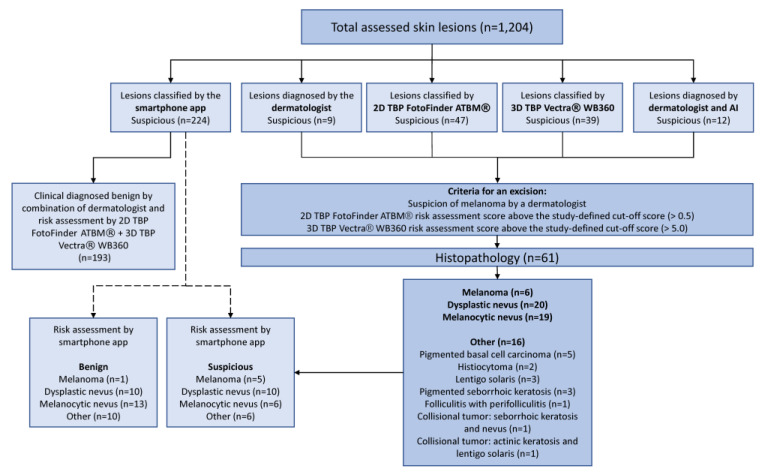
Flow chart of all included pigmented skin lesions and their histopathological outcome. AI = artificial intelligence; TBP = total body photography.

A total of 1204 pigmented skin lesions were assessed in this study (Figure 1). In 61 cases (5.1%), we performed a histopathology examination, while 1129 lesions (94%) were diagnosed to be clinically clearly benign based on the combination of a risk assessment by a dermatologist and AI-based risk scores below the cut-off (2D and 3D TBP) and had no indication for obtaining histopathology.

### 3.2. Diagnostic Accuracy and Performance of the Smartphone App SkinVision^®^

#### 3.2.1. Comparison of all Risk Assessments

**Figure 2:**
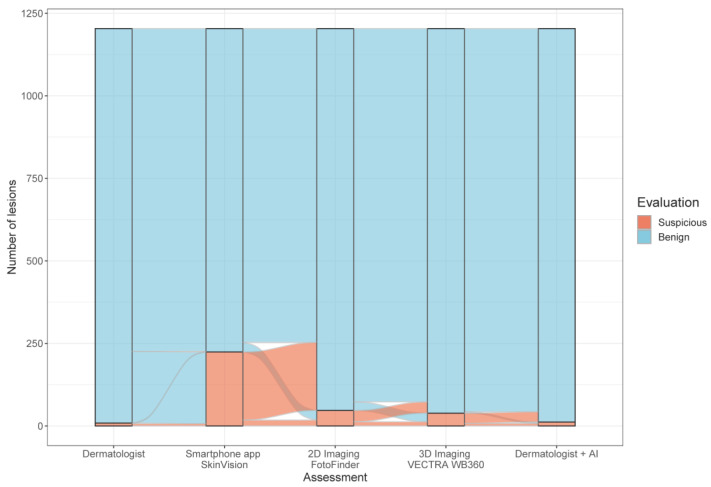
Comparison of risk assessments with the highest rate of suspected melanoma cases by the smartphone app (n = 1204).

The smartphone app SkinVision^®^ classified 980 (81%) lesions as benign and indicated an increased risk for melanoma in 224 (19%) cases, while the dermatologists diagnosed 1195 (99.3%) lesions as benign and only nine (0.7%) as suspicious. Consequently, the CE-certified app had a 27-fold higher rate of melanoma-suspicious lesions compared to dermatologists. The AI scores of the 2D and 3D CNN devices classified most lesions as benign and 47 (3.9%) lesions (FotoFinder ATBM^®^) resp. 39 (3.2%) lesions (Vectra^®^ WB360) as suspicious for melanoma (Figure 2).

Among the 224 lesions classified as suspicious by SkinVision^®^, 193 were considered clinically benign by the physician and the 2D and 3D CNN AI-risk assessment scores, whereas four pigmented skin lesions were classified suspicious by the smartphone app, dermatologists, 2D, and 3D TBP devices (Table 2, Supplementary Table S1). The knowledge of AI-based risk assessment scores did not meaningfully affect dermatologists’ classification of skin lesions, as they changed their decision towards the indication for excision in only three lesions that later turned out to be benign (Table 2).

#### 3.2.2. Diagnostic Accuracy of the Smartphone App Based on the Combination of the Dermatologist’s Evaluation plus the AI Risk-Assessment Scores of Two Independent Medical Devices

**Figure 3:**
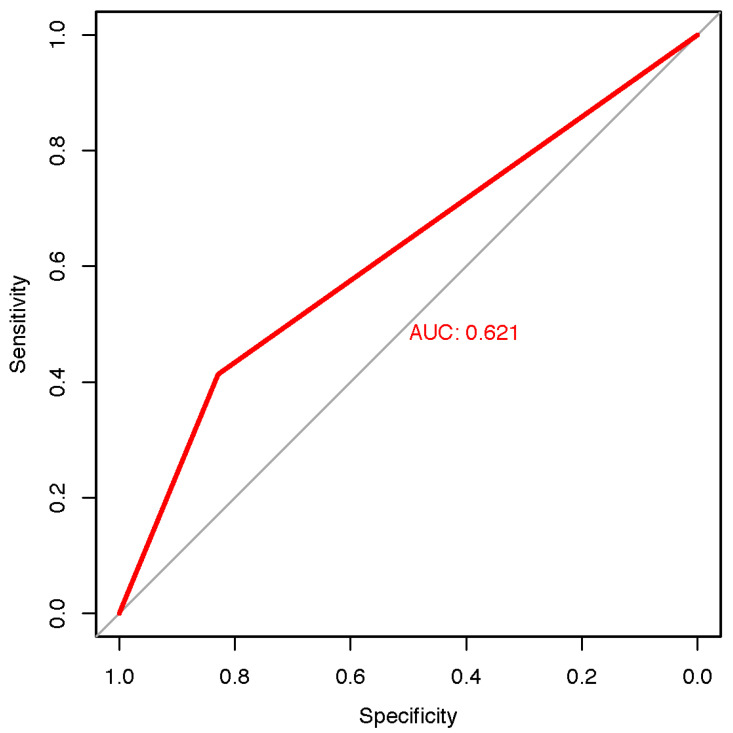
Receiver operating characteristic curve of the smartphone app in relation to the results of the combination of risk assessments by dermatologists, FotoFinder ATBM^®^, and Vectra^®^ WB360 (sensitivity: 41.3%, specificity: 82.9%); AUC = area under the curve.

Receiver operating characteristic (ROC) analysis of the classification of benign and suspicious lesions of the SkinVision^®^ app compared to the combined evaluation of FotoFinder ATBM^®^, Vectra^®^ WB360, and the dermatologists had an area under the curve (AUC) score of 0.621, sensitivity of 0.41, and specificity of 0.83 (Figure 3). Although the specificity is reasonable, the sensitivity is low, thus suggesting that the SkinVision^®^ app has poor diagnostic accuracy.

#### 3.2.3. Diagnostic Accuracy of the Smartphone App Based on Histopathology

Among 61 pigmented skin lesions examined histologically, we detected six melanomas, 19 melanocytic nevi, 20 dysplastic nevi, as well as 16 otherwise classified lesions were diagnosed (Table 3, Figure 1).

**Figure 4:**
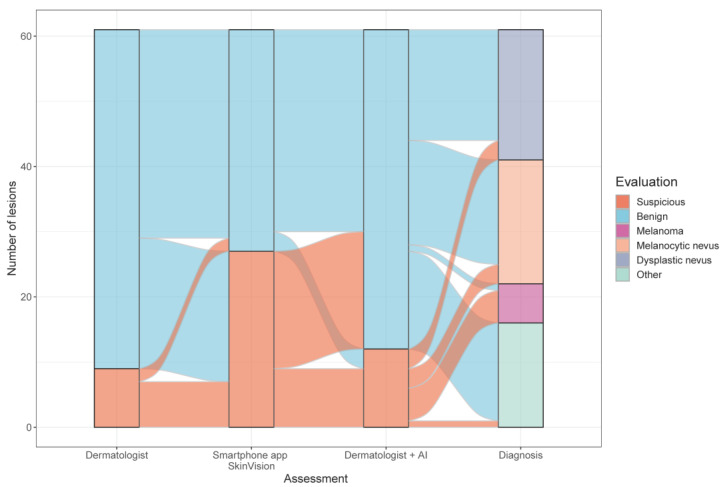
Histology and corresponding diagnosis of lesions assessed by dermatologists, smartphone app SkinVision^®^, and dermatologists and AI (n = 61).

Based on histopathology, Figure 4 represents the risk assessments of the dermatologists, the mHealth app, and the combination of AI and dermatologists. Both the SkinVision^®^ app and the 2D and 3D CNN devices indicated for 5 of 6 histological verified melanomas (83% sensitivity) an elevated score indicating suspicion of melanoma. The false-negative rate for all AI-based medical devices was 17%. The three different false-negative classified melanomas were all superficial spreading stage IA melanomas—SkinVision^®^: superficial spreading melanoma, 0.9 mm Breslow thickness, AJCC stage IA (pT1bN0M0); 3D TBP Vectra^®^ WB360: superficial spreading melanoma, 0.3 mm Breslow thickness, AJCC stage IA (pT1aN0M0); 2D TBP FotoFinder ATBM^®^: superficial spreading melanoma, 0.7 mm Breslow thickness, AJCC stage IA (pT1aN0M0). The true-negative rate for melanomas (specificity) including melanocytic nevi, dysplastic nevi, and otherwise classified diagnoses was 60.0% of the smartphone app, 63.6% of the 3D Vectra^®^ WB360 imaging device, and 40.0% of the 2D FotoFinder ATBM^®^ risk assessment tool. Dermatologists correctly identified five of six melanomas (83% sensitivity). The false-negative rate was 17%. The true-negative rate for melanomas (specificity) including melanocytic nevi, dysplastic nevi, and otherwise classified diagnoses among all dermatologists was 92.7%. The performance of the dermatologists strongly correlated with their professional experience. Hence, the true-positive rates for melanoma were greater for experts and dermatologists with intermediate experience (100% sensitivity) compared to beginners (80% sensitivity); whereas the true-negative rates were similar (93.3% resp. 92.5% specificity) (Table 3).

**Figure 5:**
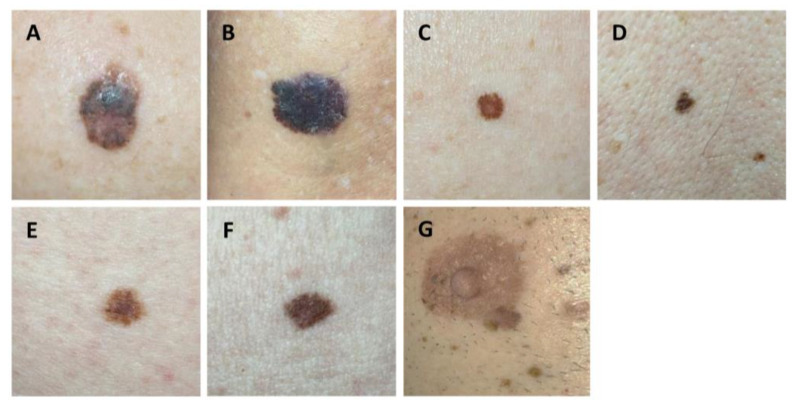
Correctly and falsely classified melanomas by the smartphone app SkinVision^®^: (**A**,**B**). True-positive classified melanoma; (**C**,**D**). False-positive classified melanoma; (**E**,**F**). True-negative classified melanoma; (**G**). False-negative classified melanoma.

Figure 5 reveals several pigmented skin lesions that were correctly and incorrectly classified as melanomas by the smartphone app SkinVision^®^.

**Figure 6:**
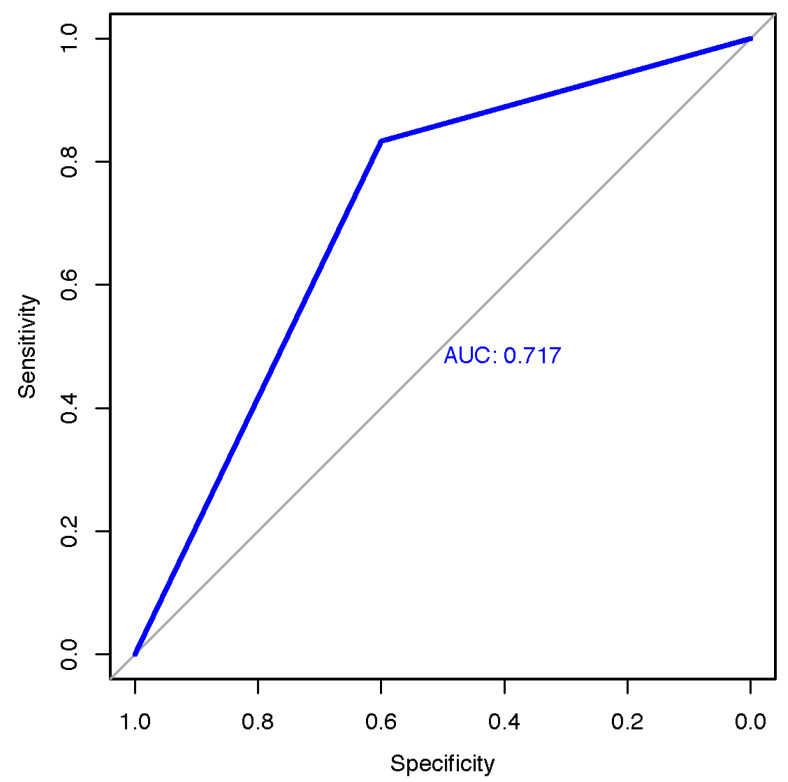
Receiver operating characteristic curve of the smartphone app in relation to the results of the histology (sensitivity of 83.3%, specificity 60.0%); AUC = area under the curve.

ROC analysis of the classification of melanoma of the SkinVision^®^ app compared to histology had an area under the curve (AUC) score of 0.717, sensitivity of 0.83, and specificity of 0.6 (Figure 6). Using histology as the gold standard, the SkinVision^®^ app had high sensitivity but mediocre specificity.

### 3.3. Patient Perspective on AI in Melanoma Screening

#### 3.3.1. Confidence in Dermatologists vs. Smartphone App

Most of the patients at high-risk for melanoma (55% (30/55)) and patients with melanoma (53% (31/59)) reported being very confident about a mole examination by a dermatologist (rating scale 10/10). In contrast, only a minority of high-risk patients (16% (9/55)) and melanoma patients (12% (7/59)) felt very safe when being investigated by a smartphone app alone. No significant difference in ratings was identified between the two risk groups (*p* < 0.9; *p* < 0.7) (Supplementary Table S2).

#### 3.3.2. Trustworthiness of the Smartphone App

**Figure 7:**
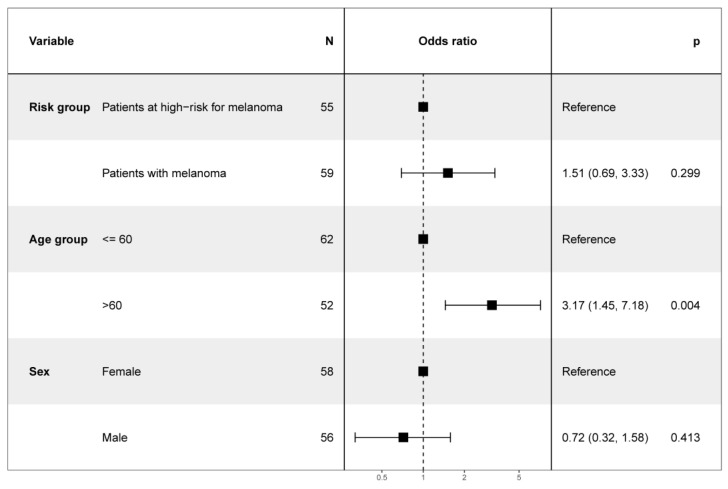
Odds ratio for variables influencing the trustworthiness of smartphones’ risk assessment in melanoma detection.

All participants primarily rated physician examination (100% (55/55) among high-risk patients, 100% (59/59) among melanoma patients) as trustworthy, and the majority did so for 2D TBP imaging (93% (51/55) resp. 88% (52/59)) and 3D TBP imaging (91% (50/55) resp. 90% (53/59)). The smartphone app was less frequently rated as trustworthy, with only 36% (20/55) among patients at high-risk for melanoma and 49% (29/59) among melanoma patients (Table 4). The age revealed a significant correlation with the evaluation of smartphones’ trustworthiness (*p* < 0.004), with older patients (>60 years old) having trusted the app three times more than younger patients (≤60 years old) (Figure 7). Neither previous melanoma vs. high-risk criteria for melanoma nor sex significantly influenced the evaluation of the trustworthiness of the smartphone app.

#### 3.3.3. Impact of AI vs. Dermatologists’ Examination on Patients’ Fear of Developing Skin Cancer

Most participants indicated that an examination by a physician reduced their fear of developing skin cancer, namely in 89% (49/55) among high-risk patients and 81% (48/59) among melanoma patients. Comparably, the 2D TBP imaging achieved the same effect in 78% (43/55) resp. 76% (45/59), and the 3D TBP device in 82% (45/55) resp. 75% (44/59). In contrast, the assessment with the smartphone app appeased fear of skin cancer in only 33% (18/55) of patients at high-risk for melanoma and in 32% (19/59) of patients with melanoma. Indeed, one high-risk patient (1.8%) even reported an increased fear of developing skin cancer by using the smartphone app (Supplementary Table S2).

#### 3.3.4. Patients’ Subjective Assessment of the Accuracy of AI vs. Dermatologists

Patients expected reliable results with the highest accuracy by both the assessment by a physician (98% (54/55) among high-risk patients, 92% (54/59) among melanoma patients) and by the 2D TBP imaging (82% (45/55) resp. 86% (51/59)) as well as the 3D TBP device (89% (49/55) resp. 88% (52/59)). Only 16% (9/55) of high-risk patients and 31% (18/59) of melanoma patients expected reliable results from the smartphone app (Supplementary Table S2). No significant differences were identified between the two risk groups in the evaluations.

#### 3.3.5. Patient Preference for Skin Cancer Screening

**Figure 8:**
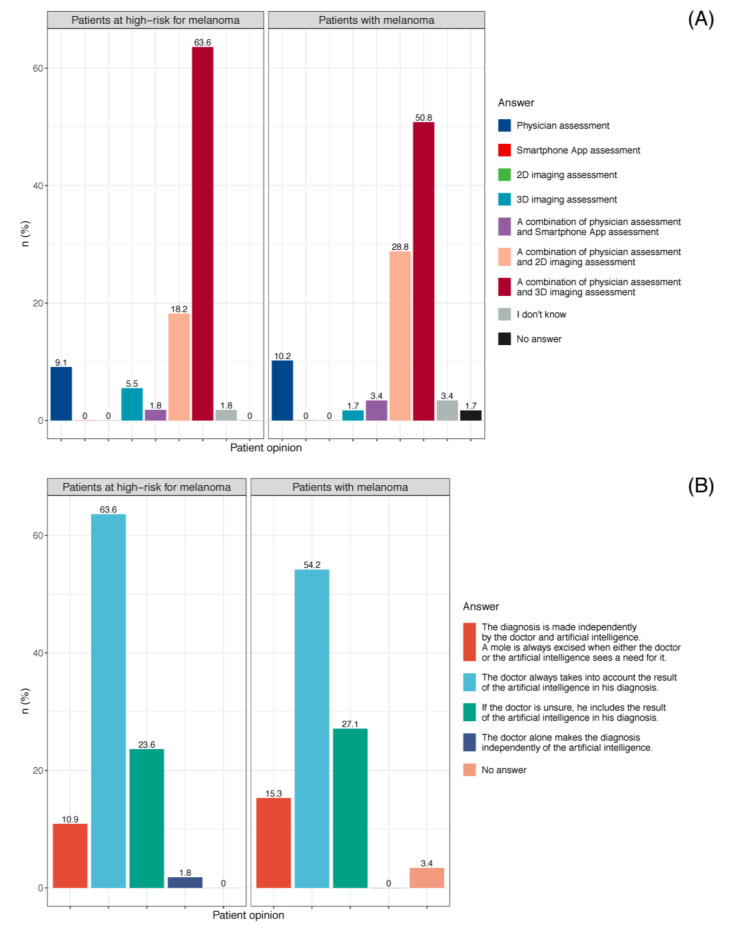
(**A**) Patient preference for mole assessment (patients at high-risk for melanoma, n = 55; patients with melanoma, n = 59); (**B**) Patient preference for AI in skin cancer screening (patients at high-risk for melanoma, n = 55; patients with melanoma, n = 59).

Both cohorts favored a combination of dermatologist and 3D TBP risk assessment for the examination of pigmented skin lesions (64% (35/55) among patients at high-risk for melanoma, 51% (30/59) among melanoma patients), while neither preferred assessment by a smartphone app alone (Figure 8A, Supplementary Table S2). The combination of dermatologist and smartphone app was favored by only 1.8% (1/55) of patients at high-risk for melanoma and 3.4% (2/59) of patients with melanoma.

Regarding patient preference for skin cancer screenings, almost all high-risk patients (98% (54/55)) and melanoma patients (95% (56/59)) indicated their belief that AI can improve a physician’s diagnostic performance. Most patients (64% (35/55) among high-risk patients, 54% (32/59) among melanoma patients) would prefer that the physicians would always consider the result of AI in their diagnosis (Figure 8B) (Supplementary Table S2).

#### 3.3.6. Dermatologists’ Perspective of Smartphone Apps for Melanoma Screening

Among the seven dermatologists, they stated in only 5.3% of the skin cancer screenings (6/114) that the smartphone app increased diagnostic confidence and in only 8.8% of the assessments (10/114) they trusted the app (Supplementary Table S3).

## 4. Discussion

### 4.1. Diagnostic Accuracy and Potential Consequences of the Smartphone App SkinVision^®^

In this prospective validation study of the CE-certified mHealth app SkinVision^®^ based on a deep learning algorithm, we observed a low diagnostic accuracy in detecting melanoma. The smartphone app’s sensitivity varied between 41.3–83.3%, the specificity between 60.0–82.9%, and the AUROC between 0.62–0.72% according to the study-defined gold standards of histopathology resp. combination of dermatologists’, 2D, and 3D risk assessments. The app’s assessment classified pigmented skin lesions 27 times more often as suspicious than the dermatologists’ evaluation. It is important to recognize that the app only provides risk stratification, not diagnosis. However, if only the app’s assessment were considered in establishing a diagnosis, this would result in an excision rate of skin lesions that is several times higher compared to the dermatologists’ evaluation. Notably, even dermatologists typically have a low threshold for excision of lesions, leading to a number needed to treat (NNT) for melanoma diagnosis of 9.60 [32]. Extrapolating our results based on this number, the SkinVision^®^ app may lead to dramatic rates of over-detection (NNT for diagnosis of melanoma 259.20) and thus needless morbidity.

Since the incorporation of AI technology has become available in smartphone apps for laypersons and thus may potentially replace a medical consultation, concerns about reliability and diagnostic accuracy are rising. Previous studies have controversially discussed the diagnostic performance of SkinVision^®^ and other smartphone apps for melanoma detection due to high variability in their diagnostic accuracy [15,33,34,35]. A prospective study evaluating the diagnostic accuracy of SkinVision^®^ compared to dermatologists’ clinical diagnoses and histological results demonstrated that the smartphone app was inferior to the diagnostic performance of dermatologists with a sensitivity of 73% vs. 88% and a specificity of 83% vs. 97% [36]. Based on the histopathological reference standard, the app achieved a slightly higher sensitivity of 83% in our study, while the specificity was lower at 60%. The high count of dysplastic nevi in our findings could have posed difficulties for the app’s algorithm in dichotomous classification (risk classification for nevus vs. melanoma), which could explain the low specificity determined on histology in our results. A recent prospective multicenter diagnostic accuracy study of SkinVision^®^ including 785 lesions indicated a sensitivity of 89.8% and a specificity of 32.9% for the app’s algorithm based on the histopathological outcome [37]. Compared to the histology, we obtained similar results for sensitivity. Nevertheless, the specificity was superior in our study. A prospective study examining the effectiveness of three melanoma apps in risk stratification of pigmented lesions also revealed low rates of sensitivity (56.8%) and specificity (50%) for the SkinVision^®^ app on iOS devices [33]. Contrary to these results, the app’s sensitivity in our study achieved 41% compared to the gold standard of the combination of dermatologists plus 2D and 3D risk assessment scores. The even lower sensitivity in our study compared with other findings could be explained by the design of our study, which represented a real-world setting with a wide variability of lesions and incorporated a high number of benign skin lesions (melanocytic and dysplastic nevi) and, comparatively, a smaller count of melanomas. However, a recent study investigating the accuracy of the smartphone app SkinVision^®^ for risk assessment of skin lesions revealed a sensitivity of 95.1% and a specificity of 78.3% for the detection of malignant or premalignant lesions, which thus indicates a promising result [20]. Nevertheless, some experts have already criticized the findings as probably overestimated due to the nature of the study design and sampling errors [21]. Considering the app’s assumed sensitivity of around 95% and specificity of approximately 80% in a low prevalence setting, for example in the UK with an incidence of 257 per 100,000 for non-melanoma skin cancer, the app would have a positive predictive value of only 1.2%, which would result in 20,000 false-positive outcomes per 100,000 users [22]. Putting our results into perspective with the published study with a high rate of false-positive scores, we also perceive the latter’s results to be overestimated and not representative.

Aside from weaknesses in the performance of the app’s algorithm, we consider macroscopic images as the major limitation in the smartphone app for classifying skin lesions. Under the current conditions, clinical close-up dermoscopic images are needed for the most accurate diagnosis both when evaluated by a physician and by an AI-based algorithm. The investigated app only refers to macroscopic images, but already available smartphone magnifying glass attachments could provide more detailed images. However, such an implementation of these attachments involves additional expensive costs for laypersons in the context of an independent screening via app and potential handling challenges.

A prospective, multicenter study including 1550 images of skin lesions acquired with smartphone and digital single-lens reflex cameras investigated the accuracy in detecting melanoma of an AI-based algorithm trained using previously published dermoscopic images [38]. The algorithm achieved an AUROC of 90.1% for biopsied lesions and 95.8% for control lesions, a sensitivity of 100%, and a specificity of 64.8% for images obtained with an iPhone. Compared to these results in dermoscopic images, the deep-learning algorithm of the smartphone app SkinVision^®^ revealed decreased diagnostic accuracy in melanoma recognition based on macroscopic images in our study with an AUROC of 62–72%. The study by Phillips et al. exemplifies that most research on the diagnostic performance of AI-based algorithm focuses on dermoscopic images, while the accuracy of macroscopic images in melanoma detection is lower and less studied.

The dermatologists in our study demonstrated high diagnostic accuracy in terms of specificity (92.7%; beginners: 92.5% vs. experts: 93.3%), which is consistent with other findings [36]. As might have been expected, the comparatively lower sensitivity of all dermatologists (83%) was dependent on professional experience (beginners: 80% vs. experts: 100%). Thus, compared with the diagnostic accuracy of the smartphone app, the dermatologists in our study performed equally in detecting melanoma regarding sensitivity. The different levels of sensitivity and specificity among the dermatologists can be explained by the years of professional experience. Dermatologists with longer professional experience have classified more pigmented skin lesions correctly according to their malignancy risk than dermatologists with less professional experience. Therefore, the error rate in the classification of melanoma is higher in novice practitioners. In contrast to the app, which classifies according to a machine-learning algorithm, the diagnostic accuracy of dermatologists seems to correlate with the number of self-classified lesions. However, the specificity of dermatologists was significantly higher compared to the app’s AI-based risk assessment. Even though our study was underpowered to reveal a relevant advantage in the combination of dermatologists and artificial intelligence, we suggest that at least beginners might benefit from AI-based risk assessments in the near future. Future studies should aim to optimize diagnostic accuracy in early melanoma detection by synergistically leveraging the high specificity of dermatologists with the diagnostic performance of AI-based technologies.

On the one hand, a significant risk posed by the low specificity of smartphone apps is over-detection, leading to misclassification of benign pigmented skin lesions as melanoma. We intend to raise awareness that false-positive lesions could lead to unnecessary surgical interventions, overextension of the health system, as well as anxiety and psychological distress for patients. On the other hand, when applying apps with low sensitivity and a high false-negative rate, underdiagnosis of melanomas in some cases is an obvious risk [34]. This might convey a false sense of security to patients and discourage them from seeing a dermatologist, which is likely to result in fatal consequences. We suggest that laypersons should use new smartphone-based screening tools with extreme caution in the absence of robust evidence-based validation studies, as they may cause potential harm to the user.

A recent systematic review about the diagnostic accuracy of algorithm-based smartphone apps for assessing skin cancer risk has criticized the fact that many diagnostic accuracy studies have weak evidence due to poor study design and thus do not support the implementation of the current apps [15]. The CE medical device classification 1 that was applied for SkinVision^®^ may be inadequate [39]. Although it is non-invasive and does not transfer energy in the body, the decision-support that the app provides can have drastic clinical consequences and thus the public is not protected sufficiently from potential risks. The FDA, on the other hand, enforces a more rigorous approval process [22] and has authorized only one app for melanoma risk stratification thus far [16]. Regulated approval of mHealth apps according to an evidence-based process is particularly important, as they influence laypersons in their decision-making process regarding further medical advice in a potentially fatal disease [22].

Although deep learning algorithms for skin cancer screening in apps are continuously evolving, we suggest with our findings that mHealth apps should not currently replace face-to-face consultation with a dermatologist.

### 4.2. The Lay and Dermatologist Perspectives on the Use of Smartphone Apps and Other AI Devices in Melanoma Screening

Acceptance by both doctors and patients is crucial for the successful use of mHealth apps in daily life. Regarding laypersons’ perspectives towards the use of smartphone apps in melanoma risk stratification, we observed a poor rating of the app’s diagnostic accuracy compared to the dermatologists’ examinations. Furthermore, the minority of patients trusted the app (49% resp. 36%). Overall, most patients preferred the combination of dermatologist and AI devices applied by physicians for skin cancer screenings and perceived AI to support dermatologists’ diagnostic performance. The sole use of the smartphone app was not favored for skin cancer screening. However, dermatologists remained more critical of the use of smartphone apps than their patients, trusting the app’s risk assessment in only 8.8% of examinations.

The generally inferior rating of the smartphone app by patients could be explained by several factors. A lack of skills as well as concerns about data use might negatively influence patients’ assessment [40]. Our results are in accordance with Sangers et al., who considered the untrustworthiness of mHealth apps and the preference for a physician instead of a smartphone app in melanoma detection as a possible barrier [26]. Contrary to our assumptions, older patients (>60 years) revealed higher confidence in the app in our findings, which might be related to greater awareness and sensitization of the disease in older age. However, other studies indicated that younger patients have more positive attitudes toward smartphone apps for melanoma detection compared to elderly patients [40,41]. Whereas in a web-based questionnaire study on the patient perspective of AI in skin cancer diagnostics, there was no significant difference between age groups [31]. Regarding the evaluation of smartphone apps’ trustworthiness for melanoma recognition, our findings demonstrated a tendency for females to have more confidence in the app compared to men. In contrast to our results, previous studies have revealed a gender-specific correlation in the assessment of mHealth apps with more males convinced of the technology [40,41]. A cross-sectional study including 200 patients represented significantly lower agreement on whether skin cancer apps could complement a personal skin examination by a physician with only 42.6%, while 98% of high-risk patients and 95% of melanoma patients in our study affirmed this thesis for the use of AI in melanoma screening [41]. Our results are in accordance with a 2020 published study examining the patient perspective of AI in skin cancer diagnosis among 298 participants, with 94% of the surveyed patients supporting the use of AI as a physician assistance system [31].

We assume that patients with a history of melanoma indicating that they are more confident in the app compared to patients at high-risk for melanoma might be based on a higher awareness of regular skin examinations due to their personal history along with an increased willingness to integrate smartphone apps into their independent skin examination. Our findings are in line with a cross-sectional study demonstrating that patients with a personal history of melanoma had a more positive attitude toward the use of smartphone apps than non-melanoma patients [41].

Most patients in our study would prefer a skin cancer screening by a combination of dermatologists and AI, especially 3D and 2D CNN devices and not mHealth apps, emphasizing the lack of smartphone app’s acceptance. Computer-aided noninvasive diagnostic systems based on dermoscopic images and neural networks have recently already achieved comparable performance compared to dermatologists under experimental conditions [42]. Our findings suggest that patients perceive great benefit from AI in skin cancer screening and that AI can assist dermatologists [30,43]. However, acceptance seems to be closely linked to the assumption that the decision-making of computer-assisted diagnostic systems is reliable, transparent, and comprehensible [31,44].

Regarding physicians’ attitudes toward the use of smartphone apps in melanoma detection, we detected an even more critical attitude compared to the participating patients. Janda et al. reported higher satisfaction when evaluating healthcare practitioners’ perspectives on store-and-forward teledermoscopy services for the diagnosis of skin cancer. 52% of the participants indicated that mobile teledermoscopy could improve the quality of their patient care, whereas, in our survey only 5.3% perceived an increase in diagnostic accuracy by smartphone apps [45]. The low confidence of dermatologists in mHealth apps could be due to feared additional workload, technical problems, or equipment costs [45]. We particularly emphasize the limitations of apps in terms of quality of images and algorithms, reliability, false reassurance for concerning lesions and unnecessary for benign lesions, patient safety and security, and additional costs as potential concerns.

Based on robust validation studies, we encourage dermatologists to inform their patients about the advantages and disadvantages of available apps for melanoma screening.

### 4.3. Strengths and Limitations

The strengths of this study are the real-world setting, the size of the included lesions (>1000), and the validation of a market-approved AI-based mHealth app. Another strength is that we did not only consider the dermatologist’s assessment as a reference standard besides histology but combined the physician’s assessment with the AI-based risk scores of the 2D and 3D TBP devices. However, due to certain limitations, the generalizability of the results should be considered with caution. Limitations of our study are that photos were taken by medically trained staff at the hospital and not by patients themselves at home. Histology was not available for all lesions due to a high number of false-positive findings reported by the app, which would have resulted in a 27-fold excessive excision rate. Another limitation is that dermatologists in combination with 2D and 3D CNN classification as a gold standard carries the risk of missing melanoma. The number of melanomas was relatively low in this study. We have only imaged pigmented skin lesions with an iOS-based smartphone and therefore could not verify possible differences in diagnostic accuracy between iOS and Android devices. Furthermore, bias due to a preselected cohort of patients at higher risk of melanoma cannot be excluded. Further comparative studies of different smartphone apps with adequate power for detecting the sensitivity and specificity of melanoma detection are needed.

## 5. Conclusions

Our study revealed a worrying over-detection of suspicious lesions by the mHealth app SkinVision^®^ as well as inferior diagnostic accuracy in melanoma detection in clinical practice. Thus, the app is not as reliable as previously advertised and indeed may potentially cause harm by making users feel uncertain and overwhelming the health system. Furthermore, the acceptance among both patients and dermatologists was scarce for the AI-based smartphone app. Although we suggest that smartphone apps should currently not replace diagnosis by a dermatologist, we still believe that AI has the potential to support physicians in grading pigmented skin lesions. Under the current circumstances, dermoscopy is needed to achieve the most accurate diagnosis by human or AI. It will be our task as dermatologists to balance the consequences of the AI decision support to reach an optimal number needed to treat, and we will need prospective studies to achieve this. Given the widespread use of smartphones, algorithm-based mHealth apps for melanoma recognition might also be a potential medium to increase awareness for melanoma screening in the lay population. However, further robust clinical evidence is crucial before including market-approved apps in self-examination by laypersons for public health benefits. We encourage healthcare professionals to advise caution and avoid potential harm as long as solid prospective evidence for a melanoma-detection app is lacking.

## Figures and Tables

**Table 1:** Characteristics of the study population and their skin cancer awareness.

Characteristic	All Patients, N = 114 ^1^	Patients with Melanoma, N = 59 ^1^	Patients at High-Risk for Melanoma, N = 55 ^1^
**Age, n (age range)**	59 (22–85)	60 (29–81)	55 (22–85)
**Sex, n (%)**			
Female	58 (51%)	32 (54%)	26 (47%)
Male	56 (49%)	27 (46%)	29 (53%)
**Risk profile, n (%)**			
Multiple melanocytic nevi (≥100) and/or dysplastic nevi (≥5) and/or positive family history for melanoma and/or diagnosis of dysplastic nevus syndrome and/or CDKN2A mutation	55 (48%)	0 (0%)	55 (100%)
Previous resected melanoma in situ or primary cutaneous melanoma	57 (50%)	57 (97%)	0 (0%)
Metastatic melanoma	2 (1.8%)	2 (3.4%)	0 (0%)
**Positive family history for melanoma,** **n (%)**	42 (37%)	11 (19%)	31 (56%)
**Frequency of skin cancer screening,** **n (%)**			
Several times per year	40 (35%)	34 (58%)	6 (11%)
Every 12 months	39 (34%)	16 (27%)	23 (42%)
Every 1–2 years	8 (7%)	4 (6.8%)	4 (7.3%)
Every 2 years	9 (7.9%)	2 (3.4%)	7 (13%)
Less than every 2 years	14 (12%)	3 (5.1%)	11 (20%)
Never	4 (3.5%)	0 (0%)	4 (7.3%)
**History of sunburns in childhood,** **n (%)**	70 (61%)	32 (54%)	38 (69%)
**Frequency of sunburns (Child), n (%)**			
Rarely (less than once per year)	44 (63%)	20 (62%)	24 (63%)
Regularly (once per year)	22 (31%)	10 (31%)	12 (32%)
Often (more than once per year)	4 (5.7%)	2 (6.2%)	2 (5.3%)
**History of sunburns in adulthood,** **n (%)**	39 (34%)	18 (31%)	21 (38%)
**Frequency of sunburns (Adult), n (%)**			
Rarely (less than once per year)	38 (97%)	18 (100%)	20 (95%)
Regularly (once per year)	0 (0%)	0 (0%)	0 (0%)
Often (more than once per year)	1 (2.6%)	0 (0%)	1 (4.8%)
**Previous tanning in the solarium,** **n (%)**	38 (33%)	13 (22%)	25 (45%)
**Usage of sunscreen (SPF), n (%)**			
SPF 6–10	2 (1.8%)	1 (1.7%)	1 (1.8%)
SPF 15–25	10 (8.8%)	3 (5.1%)	7 (13%)
SPF 30–50	64 (56%)	30 (51%)	34 (62%)
SPF 50+	38 (33%)	25 (42%)	13 (24%)

^1^ Median (Range); n (%).

**Table 2:** Risk assessments of 1204 pigmented skin lesions by the smartphone app SkinVision^®^, 2D imaging FotoFinder ATBM^®^, 3D imaging Vectra^®^ WB360, dermatologists, and dermatologists in combination with knowledge of FotoFinder ATBM^®^ and Vectra^®^ WB360 AI-scores.

Characteristic	N = 1204 ^1^
**Smartphone app SkinVision** **^®^**	
benign	980 (81%)
suspicious	224 (19%)
**2D Imaging FotoFinder ATBM^®^**	
benign	1157 (96%)
suspicious	47 (3.9%)
**3D Imaging VECTRA** **^®^ WB360**	
benign	1165 (97%)
suspicious	39 (3.2%)
**Dermatologists**	
benign	1195 (99%)
suspicious	9 (0.7%)
**Dermatologists informed about risk assessment scores by FotoFinder ATBM** **^®^ + VECTRA** **^®^ WB360**	
benign	1192 (99%)
suspicious	12 (1.0%)

^1^ n (%); AI = artificial intelligence.

**Table 3:** Diagnostic accuracy of the AI-based smartphone app SkinVision^®^, 2D imaging FotoFinder ATBM^®^, 3D imaging Vectra^®^ WB360, dermatologists, and dermatologists in combination with AI in melanoma detection based on histopathology: sensitivity and specificity.

Histopathologic Diagnosis	N	Melanocytic Nevus, N = 19 ^1^	Dysplastic Nevus, N = 20 ^1^	Melanoma, N = 6 ^1^	Other *, N = 16 ^1^
**Smartphone app SkinVision^®^**	61				
benign		13 (68%)	10 (50%)	1 (17%)	10 (62%)
suspicious		6 (32%)	10 (50%)	5 (83%)	6 (38%)
**2D imaging FotoFinder ATBM^®^**	61				
benign		7 (37%)	11 (55%)	1 (17%)	4 (25%)
suspicious		12 (63%)	9 (45%)	5 (83%)	12 (75%)
**3D imaging VECTRA** **^®^ WB360**	61				
benign		18 (95%)	9 (45%)	1 (17%)	8 (50%)
suspicious		1 (5.3%)	11 (55%)	5 (83%)	8 (50%)
**Dermatologists**	61				
benign		17 (89%)	18 (90%)	1 (17%)	16 (100%)
suspicious		2 (11%)	2 (10%)	5 (83%)	0 (0%)
*Beginner: <2 years’ work* *experience*	44	N = 15	N = 12	N = 5	N = 13
benign		14 (93%)	10 (83%)	1 (20%)	13 (100%)
suspicious		1 (6.7%)	2 (17%)	4 (80%)	0 (0%)
*Intermediate: 2–5 years’ work experience*	5	N = 2	N = 3	N = 0	N = 0
benign		1 (50%)	3 (100%)	0 (0%)	0 (0%)
suspicious		1 (50%)	0 (0%)	0 (0%)	0 (0%)
*Experts: >5 years’ work* *experience*	11	N = 2	N = 5	N = 1	N = 3
benign		2 (100%)	5 (100%)	0 (0%)	3 (100%)
suspicious		0 (0%)	0 (0%)	1 (100%)	0 (0%)
**Dermatologists informed about** **AI scores ^2^**	61				
benign		16 (84%)	17 (85%)	1 (17%)	15 (94%)
suspicious		3 (16%)	3 (15%)	5 (83%)	1 (6.2%)
*Beginner: <2 years’ work* *experience*		N = 15	N = 12	N = 5	N = 13
benign		13 (87%)	9 (75%)	1 (20%)	12 (92%)
suspicious		2 (13%)	3 (25%)	4 (80%)	1 (7.7%)
*Intermediate: 2–5 years’ work experience*		N = 2	N = 3	N = 0	N = 0
benign		1 (50%)	3 (100%)	0 (0%)	0 (0%)
suspicious		1 (50%)	0 (0%)	0 (0%)	0 (0%)
*Experts: >5 years’ work* *experience*		N = 2	N = 5	N = 1	N = 3
benign		2 (100%)	5 (100%)	0 (0%)	3 (100%)
suspicious		0 (0%)	0 (0%)	1 (100%)	0 (0%)

^1^ n (%); * Other = pigmented basal cell carcinoma, histiocytoma, lentigo solaris, pigmented seborrhoic keratosis, folliculitis with perifolliculitis, collisional tumor: seborrhoic keratosis and nevus, collisional tumor: actinic keratosis and lentigo solaris; ^2^ risk assessment scores by FotoFinder ATBM^®^ and VECTRA^®^ WB360; AI = artificial intelligence.

**Table 4:** Assessment of trustworthiness of the AI-based smartphone app SkinVision^®^, 2D imaging FotoFinder ATBM^®^, and 3D imaging Vectra^®^ WB360 compared to dermatologists.

Characteristic	N	Patients with Melanoma, N = 59 ^1^	Patients at High-Risk for Melanoma, N = 55 ^1^	*p*-Value ^2^
**The following examination was trustworthy: Smartphone app assessment**	114			0.3
Yes		29 (49%)	20 (36%)	
No		5 (8.5%)	8 (15%)	
I don’t know		23 (39%)	22 (40%)	
No answer		2 (3.4%)	5 (9.1%)	
**Dermatologist assessment**	114			
Yes		59 (100%)	55 (100%)	
No		0 (0%)	0 (0%)	
I don’t know		0 (0%)	0 (0%)	
No answer		0 (0%)	0 (0%)	
**2D TBP assessment**	114			0.3
Yes		52 (88%)	51 (93%)	
No		0 (0%)	0 (0%)	
I don’t know		7 (12%)	3 (5.5%)	
No answer		0 (0%)	1 (1.8%)	
**3D TBP assessment**	114			0.3
Yes		53 (90%)	50 (91%)	
No		0 (0%)	0 (0%)	
I don’t know		6 (10%)	3 (5.5%)	
No answer		0 (0%)	2 (3.6%)	

^1^ n (%); ^2^ Fisher’s exact test; Pearson’s Chi-squared test; TBP = total body photography.

## Data Availability

Fully anonymized data can be requested from the corresponding author.

## Supplementary Materials

The following supporting information can be downloaded at: https://www.mdpi.com/article/10.3390/cancers14153829/s1, Table S1: Combination of all risk assessments of 1204 pigmented skin lesions by the smartphone app SkinVision^®^, 2D imaging FotoFinder ATBM^®^, 3D imaging Vectra^®^ WB360, dermatologists and dermatologists in combination with knowledge of FotoFinder ATBM^®^ and Vectra^®^ WB360 AI-scores; Table S2: Patients’ preference for skin cancer screening and their assessment of the AI-based smartphone app SkinVision^®^, 2D imaging FotoFinder ATBM^®^, and 3D imaging Vectra^®^ WB360 compared to dermatologists; Table S3: Dermatologists’ perspective of smartphone apps for melanoma screening; Figure S1: Flowchart of the study procedures. CNN = Convolutional neural network, TBP = Total body photography, AI = Artificial intelligence.
