# Supplementary material for: Over-Detection of Melanoma-Suspect Lesions by a CE-Certified Smartphone App: Performance in Comparison to Dermatologists, 2D and 3D Convolutional Neural Networks in a Prospective Data Set of 1204 Pigmented Skin Lesions Involving Patients’ Perception
Source: Cancers (Basel). 2022 Aug 7;14(15):3829. doi: 10.3390/cancers14153829 (PMC9367531; doi:10.3390/cancers14153829)
Supplement: Supplementary file 1 [file cancers-14-03829-s001.zip › cancers-1815532-supplementary.pdf]

Supplementary Material

Table S1. Combination of all risk assessments of 1,204 pigmented skin lesions by the smartphone app SkinVision®, 2D imaging FotoFinder ATBM®, 3D imaging Vectra® WB360, dermatologists and dermatologist in combination with knowledge of FotoFinder ATBM®, and Vectra® WB360 AI-scores.

Characteristic	N = 1,204[1]
**overall**	
SkinVision®: benign FotoFinder ATBM®: benign Vectra® WB360: benign First Evaluation[2]: benign Second Evaluation[3]: benign	936 (78%)
SkinVision®: benign FotoFinder ATBM®: benign Vectra® WB360: benign First Evaluation: suspicious Second Evaluation: suspicious	1 (<0.1%)
SkinVision®: benign FotoFinder ATBM®: benign Vectra® WB360: suspicious First Evaluation: benign Second Evaluation: benign	14 (1.2%)
SkinVision®: benign FotoFinder ATBM®: suspicious Vectra® WB360: benign First Evaluation: benign Second Evaluation: benign	25 (2.1%)
SkinVision®: benign FotoFinder ATBM®: suspicious Vectra® WB360: suspicious First Evaluation: benign Second Evaluation: benign	2 (0.2%)
SkinVision®: benign FotoFinder ATBM®: suspicious Vectra® WB360: suspicious First Evaluation: benign Second Evaluation: suspicious	1 (<0.1%)
SkinVision®: benign FotoFinder ATBM®: suspicious Vectra® WB360: suspicious First Evaluation: suspicious Second Evaluation: suspicious	1 (<0.1%)
SkinVision®: suspicious FotoFinder ATBM®: benign Vectra® WB360: benign First Evaluation: benign Second Evaluation: benign	193 (16%)
SkinVision®: suspicious FotoFinder ATBM®: benign Vectra® WB360: benign First Evaluation: suspicious Second Evaluation: suspicious	1 (<0.1%)
SkinVision®: suspicious FotoFinder ATBM®: benign Vectra® WB360: suspicious First Evaluation: benign Second Evaluation: benign	11 (0.9%)
SkinVision®: suspicious FotoFinder: benign Vectra® WB360: suspicious First Evaluation: suspicious Second Evaluation: suspicious	1 (<0.1%)
SkinVision®: suspicious FotoFinder ATBM®: suspicious Vectra® WB360: benign First Evaluation: benign Second Evaluation: benign	7 (0.6%)
SkinVision®: suspicious FotoFinder ATBM®: suspicious Vectra® WB360: benign First Evaluation: benign Second Evaluation: suspicious	1 (<0.1%)
SkinVision®: suspicious FotoFinder ATBM®: suspicious Vectra® WB360: benign First Evaluation: suspicious Second Evaluation: suspicious	1 (<0.1%)
SkinVision®: suspicious FotoFinder: suspicious Vectra® WB360: suspicious First Evaluation: benign Second Evaluation: benign	4 (0.3%)
SkinVision®: suspicious FotoFinder ATBM®: suspicious Vectra® WB360: suspicious First Evaluation: benign Second Evaluation: suspicious	1 (<0.1%)
SkinVision®: suspicious FotoFinder ATBM®: suspicious Vectra® WB360: suspicious First Evaluation: suspicious Second Evaluation: suspicious	4 (0.3%)

[1]n (%); [2] First Evaluation = Dermatologist alone; [3] Second Evaluation = Dermatologist in combination with knowledge of 2D and 3D AI-scores

Table S2. Patients' preference for skin cancer screening and their assessment of the AI-based smartphone app SkinVision®, 2D imaging FotoFinder ATBM®, and 3D imaging Vectra® WB360 compared to dermatologists.

Characteristic	N	Patients with melanoma, N = 59[1]	Patients at high-risk for melanoma, N = 55[1]	p-value[2]
**A personal mole examination by the dermatologist makes me feel: 0 = not safe; 10 = very safe**	114			0.9
0		0 (0%)	0 (0%)	
1		0 (0%)	0 (0%)	
2		0 (0%)	0 (0%)	
3		0 (0%)	0 (0%)	
4		0 (0%)	0 (0%)	
5		0 (0%)	0 (0%)	
6		0 (0%)	0 (0%)	
7		3 (5.1%)	4 (7.3%)	
8		15 (25%)	11 (20%)	
9		10 (17%)	10 (18%)	
10		31 (53%)	30 (55%)	
**A sole examination with the smartphone app makes me feel: 0 = not safe; 10 = very safe**	114			0.7
0		3 (5.1%)	3 (5.5%)	
1		3 (5.1%)	2 (3.6%)	
2		7 (12%)	7 (13%)	
3		6 (10%)	5 (9.1%)	
4		3 (5.1%)	6 (11%)	
5		8 (14%)	6 (11%)	
6		8 (14%)	7 (13%)	
7		4 (6.8%)	7 (13%)	
8		6 (10%)	3 (5.5%)	
9		4 (6.8%)	0 (0%)	
10		7 (12%)	9 (16%)	
**Preference for mole assessment**	114			0.6
Physician assessment		6 (10%)	5 (9.1%)	
2D imaging assessment		0 (0%)	0 (0%)	
3D imaging assessment		1 (1.7%)	3 (5.5%)	
Smartphone app assessment		0 (0%)	0 (0%)	
A combination of physician assessment and 2D imaging assessment		17 (29%)	10 (18%)	
A combination of physician assessment and 3D imaging assessment		30 (51%)	35 (64%)	
A combination of physician assessment and Smartphone App assessment		2 (3.4%)	1 (1.8%)	
I don't know		2 (3.4%)	1 (1.8%)	
No answer		1 (1.7%)	0 (0%)	

Characteristic	N	Patients with melanoma, N = 59[1]	Patients at high-risk for melanoma, N = 55[1]	p-value[2]
**The following examination has reduced my fear of developing skin cancer:**	114			0.8
**Smartphone app assessment**				
Yes		19 (32%)	18 (33%)	
No		16 (27%)	11 (20%)	
I don't know		19 (32%)	19 (35%)	
No answer		5 (8.5%)	7 (13%)	
**Dermatologist assessment**	114			0.4
Yes		48 (81%)	49 (89%)	
No		6 (10%)	1 (1.8%)	
I don't know		3 (5.1%)	3 (5.5%)	
No answer		2 (3.4%)	2 (3.6%)	
**2D TBP assessment**	114			0.4
Yes		45 (76%)	43 (78%)	
No		5 (8.5%)	1 (1.8%)	
I don't know		6 (10%)	6 (11%)	
No answer		3 (5.1%)	5 (9.1%)	
**3D TBP assessment**	114			0.5
Yes		44 (75%)	45 (82%)	
No		5 (8.5%)	1 (1.8%)	
I don't know		7 (12%)	6 (11%)	
No answer		3 (5.1%)	3 (5.5%)	
**The following examination has increased my fear of developing skin cancer:**	114			>0.9
**Smartphone app assessment**				
Yes		0 (0%)	1 (1.8%)	
No		49 (83%)	44 (80%)	
I don't know		5 (8.5%)	5 (9.1%)	
No answer		5 (8.5%)	5 (9.1%)	
**Dermatologist assessment**	114			0.4
Yes		0 (0%)	0 (0%)	
No		58 (98%)	52 (95%)	
I don't know		1 (1.7%)	2 (3.6%)	
No answer		0 (0%)	1 (1.8%)	
**2D TBP assessment**	114			0.6
Yes		0 (0%)	0 (0%)	
No		55 (93%)	52 (95%)	
I don't know		3 (5.1%)	1 (1.8%)	
No answer		1 (1.7%)	2 (3.6%)	
**3D TBP assessment**	114			0.6
Yes		0 (0%)	0 (0%)	
No		55 (93%)	52 (95%)	
I don't know		3 (5.1%)	1 (1.8%)	
No answer		1 (1.7%)	2 (3.6%)	

Characteristic	N	Patients with melanoma, N = 59[1]	Patients at high-risk for melanoma, N = 55[1]	p-value[2]
**With the following examination I expect a reliable result with the highest level of accuracy:**	114			0.2
**Smartphone app assessment**				
Yes		18 (31%)	9 (16%)	
No		14 (24%)	21 (38%)	
I don't know		26 (44%)	23 (42%)	
No answer		1 (1.7%)	2 (3.6%)	
**Dermatologist assessment**	114			0.058
Yes		54 (92%)	54 (98%)	
No		0 (0%)	1 (1.8%)	
I don't know		5 (8.5%)	0 (0%)	
No answer		0 (0%)	0 (0%)	
**2D TBP assessment**	114			0.5
Yes		51 (86%)	45 (82%)	
No		0 (0%)	2 (3.6%)	
I don't know		8 (14%)	8 (15%)	
No answer		0 (0%)	0 (0%)	
**3D TBP assessment**	114			0.7
Yes		52 (88%)	49 (89%)	
No		0 (0%)	1 (1.8%)	
I don't know		7 (12%)	5 (9.1%)	
No answer		0 (0%)	0 (0%)	
**Preference for skin cancer screening if AI can distinguishes very precisely between melanoma and harmless moles**	114			0.5
If the physician is unsure, he includes the result of the artificial intelligence in his diagnosis.		16 (27%)	13 (24%)	
The diagnosis is made independently by the physician and artificial intelligence. A mole is always excised when either the physician or the artificial intelligence sees a need for it.		9 (15%)	6 (11%)	
The physician alone makes the diagnosis independently of the artificial intelligence.		0 (0%)	1 (1.8%)	
The physician always takes into account the result of the artificial intelligence in his diagnosis.		32 (54%)	35 (64%)	
No answer		2 (3.4%)	0 (0%)	

[1]n (%); [2]Fisher's exact test; Pearson's Chi-squared test; TBP = total body photography; AI = artificial intelligence

Table S3. Dermatologists' perspective of smartphone apps for melanoma screening.

Characteristic	N = 114[1] skin cancer screenings
**Increased diagnostic confidence through the smartphone app**	
Yes	6 (5.3%)
No	107 (94%)
No answer	1 (0.9%)
**Trustworthiness of the smartphone app**	
Yes	10 (8.8%)
No	103 (90%)
No answer	1 (0.9%)

[1]n (%)

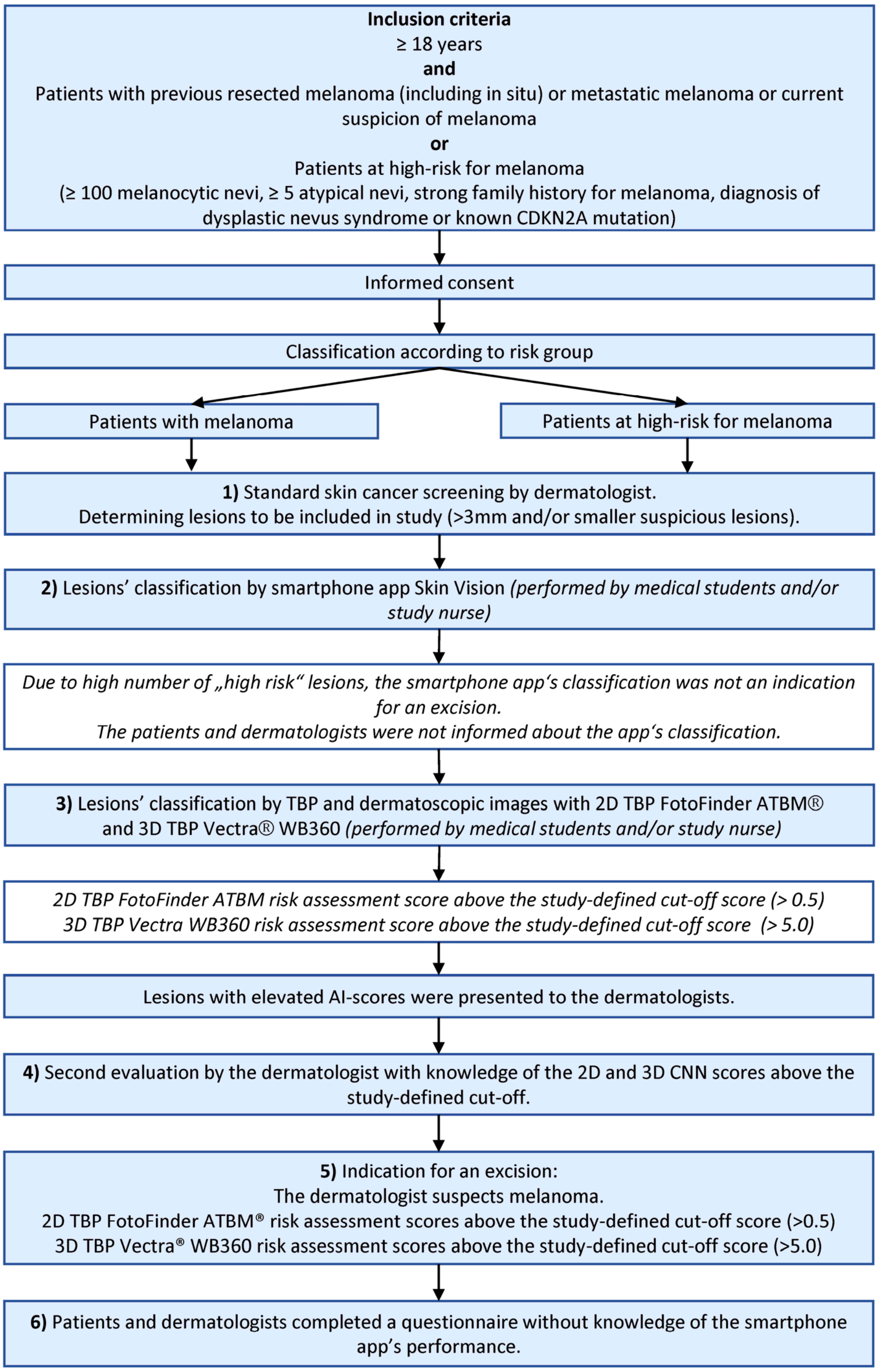

Figure S1. Flowchart of the study procedures. CNN = Convolutional neural network, TBP = Total body photography, AI = Artificial intelligence.
